# Correlates of HIV Testing among Female Sex Workers in Iran: Findings of a National Bio-Behavioural Surveillance Survey

**DOI:** 10.1371/journal.pone.0147587

**Published:** 2016-01-25

**Authors:** Mostafa Shokoohi, Mohammad Karamouzian, Razieh Khajekazemi, Mehdi Osooli, Hamid Sharifi, Ali Akbar Haghdoost, Kianoush Kamali, Ali Mirzazadeh

**Affiliations:** 1 Regional Knowledge Hub, and WHO Collaborating Centre for HIV Surveillance, Institute for Futures Studies in Health, Kerman University of Medical Sciences, Kerman, Iran; 2 Research Center for Modeling in Health, Institute for Futures Studies in Health, Kerman University of Medical Sciences, Kerman, Iran; 3 Centre for Haemostasis and Thrombosis, Skane University Hospital, Department of Translational Medicine, Lund University, Lund, Sweden; 4 Center for Disease Control (CDC), Ministry of Health and Medical Education, Tehran, Iran; 5 Department of Epidemiology and Biostatistics, University of California San Francisco, San Francisco, CA, United States of America; University of Missouri-Kansas City, UNITED STATES

## Abstract

**Introduction:**

Female sex workers (FSWs) are the second most affected population by HIV in Iran. However, their HIV testing practices are poorly understood. The aim of this study was to investigate testing and its associated factors among HIV negative FSWs.

**Materials and Methods:**

Using facility based sampling, 1005 FSWs were recruited in 14 cities of Iran in 2010. Biological and survey data were collected through dried blood spot testing and standardized risk assessment questionnaire, respectively. In this paper, the prevalence of HIV testing and its correlates were explored among 714 HIV-negative FSWs using descriptive statistics and logistic regression models.

**Results:**

Overall 65.4% had not tested in the past year. Only 27.5% had tested in the past year and received their results. FSWs who perceived themselves at risk of HIV (Adjusted Odds Ratio (AOR) = 8.35, 95% CI: 1.46, 47.6), had received free condom during past year (AOR = 3.90, 95% CI: 1.67, 9.14), started sex work at an older age (AOR_18–24_ = 2.83, 95% CI: 1.14, 7.0; AOR _>24_ = 2.76, 95% CI: 1.11, 6.84), and knew an HIV testing site (AOR = 5.67, 95% CI: 2.60, 12.4) had a significantly higher chance of having a recent HIV test result.

**Conclusions:**

Less than one third of FSWs in Iran knew their recent HIV status. Interventions to help FSWs evaluate their potential risk for HIV and integrate HIV testing services in condom distribution programs, could be viable strategies in increasing HIV testing uptake among FSWs. Health policy makers should also try to de-stigmatize HIV testing, identify the barriers to HIV testing, and make HIV testing sites more visible to FSWs.

## Introduction

HIV epidemic in Iran has mainly affected people who inject drugs (PWID), female sex workers (FSWs), partners of PWID, and prisoners [1]. Although the HIV epidemic in Iran is largely driven by injection drug use, heterosexual transmission of HIV has been constantly on the rise [1–3]. Over 28,000 people living with HIV (PLHIV) have been identified and registered by the end of 2014; a figure well below the estimated number of over 76,000 PLHIV [1, 4]. Out of all registered HIV cases in Iran, 11.7% are women, 14.1% of whom have contracted HIV through sexual contact [1, 5].

FSWs are at an increased risk of HIV infection and serve as a bridging group in transmitting the virus to the general population. Global estimates suggest that the odds of HIV infection amongst FSWs in low and middle income countries is 13.5 times that of other women of reproductive age [6]. As FSWs have recently been acknowledged as a population most-at-risk of HIV in Iran, data on FSWs remains relatively limited. It was estimated that as of 2013, an approximate number of 30,000–60,000 FSWs lived in the country with an overall HIV prevalence of 4.5% [7]; a figure likely to be an underestimate [8]. Currently, several centres including special centres for vulnerable women, drop-in centres (DIC), shelters, and voluntary counselling and testing (VCT) centres throughout the country provide FSWs with services such as basic sexual and reproductive health care, free HIV testing and counselling, and methadone maintenance therapy [1, 9, 10].

As repeated HIV testing is highly recommended for all key populations including FSWs [11], Iran has recently scaled up its HIV testing, treatment and care services among FSWs in order to increase their HIV testing practices. However, profound stigma against FSWs at different social and organizational levels on top of their limited awareness of HIV testing sites and services, create significant barriers towards routine HIV testing practices amongst FSWs in Iran [10, 12, 13]. For example, despite the availability of free HIV testing for FSWs, less than half of them had ever tested for HIV in 2010 [1, 10]. In order to increase HIV testing rates among FSWs in Iran, it is essential to understand the determinants of their testing behaviour. Using data from the first round of a national survey among FSWs conducted in 2010, this paper aims to assess the HIV testing prevalence and its correlates among FSWs in Iran.

## Materials and Methods

### Study Design

The first Bio-Behavioural Surveillance Survey (BBSS) among FSWs in Iran was performed during April and July 2010. Details of methodology and sampling strategies of the survey are described elsewhere [10]. In brief, data was collected through face-to-face interviews across 14 cities in 21 facilities catered towards vulnerable women (e.g., FSWs, partners of PWID, and women who inject drugs). Survey sites were selected to include a geographically representative cross section of different regions of Iran according to the suspected level of HIV prevalence among FSWs. Facilities were chosen based on the advice from the local health department surveillance team and Ministry of Health, on the capacity of staff to consent, enrol, and interview with the participants, the resources of the facility to implement the survey, and the number of FSWs registered and receiving services in each facility.

### Data Collection

A convenience sample of 30–45 eligible participants was taken at each recruitment site. Eligible participants were FSWs who were ≥18 years, had exchanged sex for money, drugs, or goods at least once during the year preceding the survey, had a history of practising sex work for at least 6 months, held Iranian citizenship, were residing in the city of the survey, and provided consent for participation. Data collection was anonymous and no names or personal identifying information was recorded during recruitment, informed consent, interview, or HIV testing. Demographic and behavioural data including sexual and reproductive health, HIV testing, knowledge of sexual prevention of HIV, sexually transmitted infections, and risky behaviours was collected through a pilot-tested risk assessment questionnaire. As rapid HIV testing was not available at the time of the survey, blood samples were taken using the dried blood spot (DBS) technique [14] and were tested for HIV antibodies by ELISA. Participants could receive their HIV test results, post-test counselling, and referrals from the local testing and counselling centre by providing the unique study identification code. For the purpose of this analysis, FSWs with positive HIV test in this study were excluded due to their potentially different testing practices.

We initially approached 1005 women; 33 were excluded for not meeting one of the eligibility criteria and 100 were excluded due to the low quality of data collection in one province (based on our provincial supervisor’s advice). Out of the 872 eligible cases, 55 denied consent to HIV test. Out of the remaining 817 samples, 69 were excluded due to low quality of blood samples. We restricted our sample to HIV-negative FSWs (excluding 30 HIV-positive cases and four cases that did not remember their last HIV testing date) for the present analysis. The final analytic sample included 714 participants.

### Dependent Variable: Having a Recent HIV Test Result

The current analysis explores the determinants of having tested for HIV and received the results in the past year (recent HIV test result from now on) among FSWs. Participants were asked “Have you ever tested for HIV in the past year?” Positive responses were followed by “Did you receive your test results the last time you tested for HIV?” As UNAIDS defines HIV testing indicator among female sex workers as “number of respondents who have been tested for HIV and received the results in last 12 months as numerator and total number of respondents as denominator” [15], HIV testing was treated as a binary variable and the responses were coded as yes, or no. The ‘yes’ category included those who had tested in the past year and received their results. The ‘no’ category was a pooled category of those who had never tested for HIV in the previous year or had tested but not received their results.

### Independent Variables

Variables evaluated as potential predictors included age at interview (≤24, 25–34, or ≥35 years old), age at sex work debut (<18, 19–24, or >24 years old), participants’ highest level of education (≤primary school, middle school, or ≥high school), marital status (single, married, or others), having other sources of income than sex work (yes or no), ever injected drugs (yes or no), age at first drug use (<16 or 16–18, or >18 years old), ever had sex with a non-paying partner (yes or no), having sufficient knowledge of sexual prevention of HIV (yes or no), history of selling sex in a brothel (yes or no), self-perceived risk of HIV (yes or no), received free condoms during last year (yes or no), healthcare-related HIV stigma (yes or no), and knowing an HIV testing site (yes or no).

### Statistical Analysis

Frequencies and descriptive statistics were computed for all main variables. As FSWs were recruited from different sites in the country, the recruitment sites were considered as sampling units and their clustering effects were adjusted using Stata survey commands. All estimates were weighted based on inverse probability weight to adjust for nonresponse. Bivariate and multivariable logistic regression models were constructed to investigate the determinants of having a recent HIV testing result among FSWs. Crude and adjusted odds ratios (AORs) as well as 95% confidence intervals (CI) were reported. Variables with a P-value <0.2 in the bivariate analysis were entered into the multivariable regression model. Variables found to be independently associated with HIV testing based on the literature (i.e., self-perceived risk of HIV, and age at sex work debut) were also included in the multivariable model. We also considered the interaction effect of self-perceived risk of HIV on the association of other variables with HIV testing. As these interaction terms appeared to improve the overall fitness of the model, they were included in the final multivariable model. Stata version 11 (Stata Corp.) was used throughout the analysis and P-values less than 0.05 were considered statistically significant.

### Ethical Considerations

Ethical issues in this survey included the guarantee of the participants’ confidentiality and verbal informed consent for the behavioural interview and blood sample collection. Given the criminalized nature of sex work in Iran and the low level of literacy among a considerable subset of the participants, which pose challenges to written informed consent, FSWs interviewed provided verbal informed consent. Interviewers explained the informed consent form to the participants and signed on the data collection forms to confirm that they had obtained verbal informed consent for all eligible FSWs. Participants were also briefed about the voluntary nature of their participation, objectives of the survey, the incentives provided, and the anonymity of all collected data. Participants’ refusal to participate in the survey did not interfere with or influence the services provided to them. The ethics committee of the Kerman University of Medical Sciences approved the study protocol and waived the need for written informed consent (2010—no. 90/122).

## Results

The characteristics of HIV-negative and HIV-positive participants are presented in Table 1. Only HIV-negative subgroup results are discussed here. Most participants (40.5%) were 25–34 years old and 38.4% were married at the time of the survey. About 47% of the participants had completed primary school or less and only 35.7% had a source of income other than sex work. Around half of the participants (48.0%) had started selling sex after the age of 24 and the median age for sex work debut was 24 years. Ever drug injection was reported by 13.0% and most (61.0%) started drug use after 18 years. About 61% of FSWs had received free condoms from harm reduction services during the last 12 months. Sufficient Knowledge of sexual prevention of HIV was observed in 75.3% whereas only 53.1% perceived themselves at risk of HIV. Knowing an HIV testing site was reported by 62.4% of the participants. Around 58% had reported sex with a non-paying partner, and 33.7% had ever worked in brothels. Around 12% of FSWs reported having ever experienced stigma in healthcare settings.

**Table 1:** Characteristics of the FSWs in Iran’s first national bio-behavioural surveillance survey (2010).

Variables	HIV-negative FSWs (n = 718)	HIV-positive FSWs (n = 30)	P-value
Age			
≤24	182 (25.3)	0 (0)	0.086
25–34	289 (40.5)	17 (54.9)	
≥35	241 (34.2)	13 (45.1)	
Marital status			
Single	134 (17.5)	2 (8.5)	0.366
Married	279 (38.4)	11 (33.2)	
Others (widow, divorced, sigheh*)	302 (44.1)	17 (58.3)	
Education			
Primary school and less	323 (46.5)	16 (51.9)	0.800
Middle school	184 (25.4)	6 (19.7)	
High school and above	211 (28.1)	8 (28.4)	
Other sources of income than sex work	236 (35.7)	16 (56.4)	0.037
Age at sex work debut			
<18	147 (24.1)	4 (19.5)	0.418
18–24	177 (27.9)	3 (14.1)	
>24	329 (48.0)	20 (66.4)	
Injection drug use (ever)	92 (13.0)	10 (35.7)	0.002
Age at the first drug use			
<16	89 (18.4)	4 (23.8)	0.581
16–18	103 (20.6)	6 (25.4)	
>18	320 (61.0)	13 (50.8)	
Receiving free condoms (last year)	432 (60.9)	24 (73.5)	0.319
Knowledge of sexual prevention of HIV	555 (75.3)	25 (79.5)	0.695
Self-perceived risk of HIV	293 (53.1)	24 (87.6)	0.0008
Knowing an HIV testing site	420 (62.4)	27 (91.0)	0.004
Sex with a non-paying partner (ever)	410 (58.5)	17 (62.3)	0.737
Selling sex in brothel (ever)	236 (33.7)	8 (25.1)	0.313
Healthcare-related stigma	79 (11.5)	5 (15)	0.694

* Sigheh: The practice of temporary marriage.

FSWs’ age was significantly correlated with their age at sex work debut (P-value = 0.0001). While most FSWs ≤24 years old (58%) had started sex work before 18 years of age, only 13% and 6% of FSWs aged 25–34 and ≥35 had started selling sex before 18, respectively. Moreover, most FSWs (57% of 25–34 and 83% of ≥35 age group) had started sex work after the age of 24.

Those participants (n = 30) who were living with HIV were significantly more likely to have other sources of income than sex work (56.4% vs. 35.7%, P-value = 0.037), have ever injected drugs (35.7% vs. 13.0%, P-value = 0.002), considered themselves at risk of HIV (87.6% vs. 53.1%, P-value = 0.0008), and knew an HIV testing site (91.0% vs. 62.4%, P-value = 0.004) compared to HIV-negative FSWs **(Table 1)**.

Among the HIV-negative subgroup, 72.5% did not have a recent HIV test result (65.4% had never tested for HIV in the past year, 7.1% had tested over the past year but not received their result), and 27.5% had tested in the past year and received their test result. Overall, 76.7% of 25–34 year-old FSWs, 73% of married FSWs, 77.4% of those with ≤primary school educations, and 74.6% of those without other sources of income than sex work did not have a recent HIV test result. Moreover, 77.2% of those who started selling sex at <18 years, 60.7% of those with a history of drug injection, 73.1% of those who started drug use after 18, 72.4% of FSWs who had sex with a non-paying partner, and 72.5% of those with a history of working in brothels did not report having a recent HIV test result. Finally, 69.6% of those with a sufficient knowledge of sexual prevention of HIV, 71.9% of those with a self-perceived risk of HIV, 64.8% of those who received free condoms in the previous year, 59.2% of those who knew an HIV testing site, and 70.1% of those who had experienced stigma in healthcare settings did not report testing for HIV and receiving their results **(Table 2)**.

**Table 2:** Recent HIV testing among different subgroups of FSWs in Iran’s first national bio-behavioural surveillance survey (2010).

Variables	No recent HIV test result N (%)	Recent HIV test result N (%)	P-value
Overall	527 (72.5)	187 (27.5)	
Age			
≤24	137 (74.4)	44 (25.6)	0.174
25–34	223 (76.7)	65 (23.3)	
≥35	162 (66.7)	77 (33.3)	
Marital status			
Single	100 (72.9)	33 (27.1)	0.990
Married	209 (73.0)	67 (27.0)	
Others (widow, divorced, sigheh*)	217 (72.5)	85 (27.5)	
Education			
Primary school and less	249 (77.4)	70 (22.6)	0.073
Middle school	131 (70.0)	53 (30.0)	
High school and above	147 (67.1)	64 (32.9)	
Other sources of income than sex work			
Yes	165 (68.6)	71 (31.4)	0.308
No	341 (74.6)	113 (25.4)	
Age at sex work debut			
<18	113 (77.2)	34 (22.8)	0.496
18–24	130 (70.6)	47 (29.4)	
>24	235 (70.9)	94 (29.1)	
Injection drug use (ever)			
Yes	59 (60.7)	33 (39.3)	0.018
No	468 (74.4)	154 (25.6)	
Age at the first drug use			
<16	61 (68.3)	28 (31.7)	0.416
16–18	81 (80.0)	21 (20.0)	
>18	230 (73.1)	89 (26.9)	
Sex with a non-paying partner (ever)			
Yes	304 (72.4)	104 (27.6)	0.937
No	222 (72.8)	83 (27.2)	
Selling sex in brothel (ever)			
Yes	172 (72.5)	63 (27.5)	0.954
No	337 (72.3)	120 (27.7)	
Knowledge of sexual prevention of HIV			
Yes	397 (69.6)	155 (30.4)	0.027
No	130 (81.6)	32 (18.4)	
Self-perceived risk of HIV			
Yes	223 (71.9)	70 (28.1)	0.615
No	174 (68.9)	86 (31.1)	
Receiving free condoms (last year)			
Yes	281 (64.8)	147 (35.2)	0.0002
No	237 (85.6)	37 (14.4)	
Knowing an HIV testing site			
Yes	253 (59.2)	166 (40.8)	<0.0001
No	252 (92.4)	19 (7.6)	
Healthcare-related stigma			
Yes	56 (70.1)	23 (29.9)	0.788
No	471 (72.9)	164 (27.1)	

* Sigheh: The practice of temporary marriage.

In the bivariate analysis, having a recent HIV test result was not significantly associated with age, marital status, educational level, having other sources of income than sex work, age at sex work debut, age at first drug use, history of working in a brothel, self-perceived risk of HIV, sex with a non-paying partner, and experience of stigma at healthcare settings. Conversely, the odds of having a recent HIV test result was significantly higher among those who knew a place for HIV testing (OR = 7.82, 95% CI: 3.62, 16.90), had received free condom in the last 12 months (OR = 3.32, 95% CI: 1.87, 5.87), had sufficient knowledge of sexual prevention of HIV (OR = 1.94, 95% CI: 1.08, 3.47), and had ever injected drugs (OR = 1.87, 95% CI: 1.11, 3.16).

In the multivariable model, knowing an HIV testing site (AOR = 5.67, 95% CI: 2.60, 12.4), receiving free condom during last 12 months (AOR = 3.90, 95% CI: 1.67, 9.14), self-perceived risk of HIV (AOR = 8.35, 95% CI: 1.46, 47.6), and age at sex work debut (AOR_18–24_ = 2.83, 95% CI: 1.14, 7.0; AOR_>24_ = 2.76, 95% CI: 1.11, 6.84) remained significant predictors of having a recent HIV test result **(Table 3)**. We also found that self-perceived risk of HIV modified the effects of knowledge of sexual prevention of HIV, age at sex work debut, and receiving free condom on having a recent HIV test result. Older FSWs who did not perceive themselves at risk of HIV were more likely to have a recent HIV test result (AOR_18–24_ = 1.99, 95% CI: 0.85, 4.66; AOR_>24_ = 2.27, 95% CI: 1.05, 4.91). Likewise, FSWs with sufficient knowledge of sexual prevention of HIV who did not perceive themselves at risk of HIV were more likely to have tested for HIV and received their results (AOR = 2.36, 95% CI: 1.03, 5.41). Lastly, FSWs who received free condoms during last 12 months and did not perceive themselves at risk of HIV were more likely to have an HIV test result (AOR = 4.44, 95% CI: 2.36, 8.37).

**Table 3:** Predictors of having a recent HIV test results among FSWs in Iran’s first national bio-behavioural surveillance survey (2010).

Variables	Crude OR(95% CI)	P-value	Adjusted OR*(95% CI)	P-value
Age				
≤24	Ref	-	-	-
25–34	0.91 (0.54, 1.52)	0.702	-	-
≥35	1.49 (0.77, 2.85)	0.220	-	-
Marital status				
Single	Ref	-	-	-
Married	0.99 (0.44, 2.30)	0.999	-	-
Others (widow, divorced, sigheh**)	1.02 (0.57, 1.82)	0.942	-	-
Education				
Primary school and less	Ref	-	Ref	-
Middle school	1.47 (0.92, 2.32)	0.097	1.62 (0.86, 3.1)	0.131
High school and above	1.67 (0.98, 2.83)	0.057	1.25(0.73, 2.15)	0.400
Other sources of income than sex work				
No	Ref	-	-	-
Yes	1.35 (0.76, 2.41)	0.281	-	-
Age at sex work debut				
<18	Ref	-	Ref	-
18–24	1.30 (0.77, 2.21)	0.308	2.83 (1.14, 7.0)	0.026
>24	1.24 (0.70, 2.23)	0.445	2.76 (1.11, 6.84)	0.030
Injection drug use (ever)				
No	Ref	-	Ref	-
Yes	1.87 (1.11, 3.16)	0.021	1.64 (0.84, 3.22)	0.136
Age at the first drug use (year)				
<16	Ref	-	-	-
16–18	0.66 (0.27, 1.33)	0.203	-	-
>18	0.71 (0.3, 1.7)	0.392	-	-
Receiving free condoms (last year)				
No	Ref	-	Ref	-
Yes	3.32 (1.87, 5.87)	<0.0001	3.90 (1.67, 9.14)	0.003
Selling sex in brothel (ever)				
No	Ref	-	-	-
Yes	0.97 (0.63, 1.48)	0.902	-	-
Knowledge of sexual prevention of HIV				
No	Ref	-	Ref	-
Yes	1.94 (1.08, 3.47)	0.028	2.15 (0.82, 5.63)	0.111
Self-perceived risk of HIV				
No	Ref	-	Ref	-
Yes	0.74 (0.41, 1.35)	0.318	8.35 (1.46, 47.6)	0.019
Knowing an HIV testing site				
No	Ref	-	Ref	-
Yes	7.82 (3.62, 16.90)	<0.0001	5.67 (2.60, 12.4)	< 0.0001
Sex with a non-paying partner (ever)				
No	Ref	-	-	-
Yes	1.02 (0.60, 1.72)	0.943	-	-
Healthcare-related stigma				
No	Ref	-	-	-
Yes	1.15 (0.39, 3.36)	0.793	-	-

*Variables with a P-value less than 0.2 in the univariate analysis and variables found to be independently associated with HIV testing based on the literature (i.e., self-perceived risk of HIV, and age at sex work debut) were entered into multivariable analysis

**Sigheh: The practice of temporary marriage.

## Discussion

The findings of the first national survey of FSWs in Iran indicates that less than one-third of FSWs had ever tested for HIV and only one-fourth had tested for HIV in the past year and knew their status. HIV testing was associated with self-perceived risk of HIV, receiving free condom in the past year, older age at sex work debut, and knowing an HIV testing site. Low HIV testing frequency among our participants is comparable with previous studies on FSWs in the major cities of Tehran (25%) [16] and Shiraz (45.2%) [17]. In the context of Middle East and North Africa (MENA) region, HIV testing prevalence among our sample was lower than Libyan FSWs who 38.6% had recent HIV test results [18]. However, it was relatively higher than ever testing frequencies of FSWs in Sudan ranging between 4–24% [19].

Despite the social taboos around FSWs in the country, Iranian health policy makers have recognized FSWs’ healthcare and social needs and catered several care and prevention services (e.g., free HIV testing) towards them. Increasing educational interventions for Iranian FSWs in the past few years, have shown promise in increasing their knowledge of HIV transmission and prevention [1]; a finding that was also supported in our study. While higher levels of HIV knowledge have been significantly associated with higher testing rates [20], a growing body of evidence suggests that sufficient knowledge of HIV does not necessarily translate into reduced risky behaviours [21]. Despite FSWs’ fair HIV knowledge, most of those without a history of HIV testing were unaware of available HIV testing sites. Making HIV testing sites more visible to those who are at risk or want to be tested for HIV might help with earlier diagnosis of infected individuals and increase the frequency of HIV testing among this sub-population.

Receiving free condoms from harm reduction centres has been reported as one of the main reasons of Iranian FSWs’ referrals to these sites [22]. Our study showed that receiving free condoms was strongly associated with higher HIV testing frequency among FSWs. Accessing healthcare services has been consistently reported as a significant predictor of HIV testing among key populations. Fortunately, Iran has recently shifted its policy in HIV testing from VCT to provider initiated HIV testing for key populations; an intervention that could improve HIV testing coverage among FSWs. However, these services at the Ministry of Health are not fully integrated into their parallel harm reduction programs provided by State Welfare Organization. Furthermore, outreach services, such as mobile HIV testing efforts have shown promise in increasing HIV testing practices among FSWs in other settings and could be a viable option in the context of Iran [23–25]. Studies have suggested that further mobile outreach activities are needed to continually promote HIV testing among FSWs, in particular those with restricted access to health and harm reduction services [26]. However, their acceptability among FSWs in Iran is not yet fully understood and calls for further research.

Additionally, FSWs’ self-perceived risk of HIV that has been also associated with higher HIV testing practices [19, 20], remained low among our participants. In our study, FSWs who perceived themselves at risk of HIV were around ten times more likely to have a recent HIV test result. Self-perceived risk also appeared as a modifying factor in the association of knowledge of sexual prevention of HIV, age at sex work debut, and receiving free condom with having a recent HIV test result. Therefore, HIV prevention programs should be redesigned in a more comprehensive way to help translate FSWs’ knowledge of HIV to reduced risky behaviour practices.

Structural and individual stigma associated with HIV and sex work have been reported as significant barriers against HIV testing efforts catered towards FSWs across MENA [27]. UNAIDS reports suggest that only 4% of the overall HIV tests conducted between 1995 to 2008 have been for key populations at risk of HIV [28]. While experiences of HIV-related stigma in healthcare settings was not a significant predictor of HIV testing practices among FSWs in our study, criminalization of sex work, concerns about the confidentiality of HIV testing, healthcare provider’s stigmatizing attitudes towards FSWs, and lack of rapid HIV testing across all HIV testing sites have been reported as some of the barriers to HIV testing among FSWs in Iran [1, 21, 22, 29].

Older age at sex work debut was significantly associated with having a recent HIV test result. We observed a significant correlation between FSWs’ age and age at sex work debut in a way that younger FSWs had started sex in very young ages (<18) and older FSWs had started sex after 24 years of age. Girls who start sex work at an earlier age are often less educated about HIV (compared to older FSWs) and therefore less likely to seek testing services and more likely to be at risk for HIV infection [30–33]. Currently Iranian youth’s primary official exposure to sexual health information including HIV, has been either through very limited topics in school curricula and pre-marital mandatory courses [34]. As most FSWs in our study have up to high school education, their limited knowledge on HIV could be addressed through introducing HIV-related educational materials to school curricula. This lower HIV testing uptake among younger FSWs could also be associated with existing barriers to HIV testing among youth including their lower access to healthcare services, higher confidentiality concerns, anticipated stigma, and lower HIV risk perceptions.

We would like to acknowledge the limitations of our findings. The data presented here was collected in 2010 and may not completely reflect current practices of FSWs; however, publishing our findings just became feasible recently considering the social and political context of Iran. Given the very limited data on Iranian FSWs’ HIV testing practices, we believe our findings of the only national survey of FSWs to date, will still have important implications for both research and policy. Moreover, our cross-sectional study design limits causal inferences and the voluntary nature of participation and self-reported data do not rule out potential selection or social desirability biases associated with such behavioural surveys. Our non-random sample may also limit the generalizability of our findings to high socioeconomic FSWs and those who do/can not use the facilities serving FSWs (outreach FSWs). They might be likely to have different testing practices and seek testing in private sectors (high socio-economic FSWs) or not seek testing at all (outreach FSWs). Nonetheless, while achieving a gold-standard survey methodology to provide a very representative sample of FSWs in Iran continues to be a challenging practice, efforts were made to reduce potential biases by recruiting a large national sample size as well as engaging local organizations and staff as well as experienced interviewers. Further qualitative studies to assess the barriers to HIV testing among FSWs in Iran are required to develop an effective model of HIV testing and promoting HIV testing rate in this underserved population. We were also not able to collect data on variables such as structural stigma, distance from testing centre, and association with community support groups which could be beneficial to consider in future surveys.

## Conclusions

All in all, such a low HIV testing rate among FSWs is very concerning and can have serious consequences for the healthcare system. This calls for strategies to scale up rapid HIV testing, improve FSWs’ perceptions of HIV risk, increase the visibility of HIV testing sites, destigmatize HIV testing, and identify the barriers to HIV testing in order to help improve HIV testing uptake among FSWs.
